# The Correlation of *PBK* Expression with an Immune-Activated Tumor Microenvironment and Outcome in Colorectal Cancer

**DOI:** 10.3390/cancers18030482

**Published:** 2026-01-31

**Authors:** Hiroshi Sawaguchi, Takeshi Uehara, Mai Iwaya, Shiho Asaka, Tomoyuki Nakajima, Shotaro Komamura, Shunsuke Imamura, Yugo Iwaya, Shinsuke Sugenoya, Masato Kitazawa, Yuji Soejima, Hiroyoshi Ota, Tadanobu Nagaya

**Affiliations:** 1 Department of Medicine, Division of Gastroenterology and Hepatology, Shinshu University School of Medicine, Matsumoto 390-8621, Japan; hiroshi-s@shinshu-u.ac.jp (H.S.); imamurash@shinshu-u.ac.jp (S.I.); yiwaya@shinshu-u.ac.jp (Y.I.); nagaya@shinshu-u.ac.jp (T.N.); 2 Department of Laboratory Medicine, Shinshu University School of Medicine, Matsumoto 390-8621, Japan; mkatou@shinshu-u.ac.jp (M.I.); sasaka@shinshu-u.ac.jp (S.A.); tnakajima@shinshu-u.ac.jp (T.N.); show97taro@shinshu-u.ac.jp (S.K.); hohta@shinshu-u.ac.jp (H.O.); 3 Department of Laboratory Medicine and Pathology, Life Science Research Center, Nagano Children’s Hospital, Azumino 399-8288, Japan; 4 Department of Surgery, Division of Gastroenterological, Hepato-Biliary-Pancreatic, Transplantation and Pediatric Surgery, Shinshu University School of Medicine, Matsumoto 390-8621, Japan; s-shinsuke@shinshu-u.ac.jp (S.S.); kita118@shinshu-u.ac.jp (M.K.); ysoejima@shinshu-u.ac.jp (Y.S.); 5 Department of Clinical Laboratory Sciences, Shinshu University School of Medicine, Matsumoto 390-8621, Japan

**Keywords:** *PBK*, colorectal cancer, tumor microenvironment, prognosis, immune microenvironment

## Abstract

**Background/Objectives**: PDZ-binding kinase (PBK) regulates mitosis, but its clinical significance and cellular localization in colorectal cancer (CRC) remain unclear. We evaluated *PBK* expression in CRC tissues and examined its association with clinicopathological features, immune contexture, and outcomes. **Methods**: *PBK* expression was assessed by RNA in situ hybridization in tumors from 246 CRC patients. Associations with TNM stage, vascular invasion, MMR status (dMMR/pMMR), immune cell infiltration, and stromal programmed death-ligand 1 (PD-L1) were analyzed. Overall survival (OS) and recurrence-free survival (RFS) were evaluated using Kaplan–Meier and Cox models. Public single-cell RNA sequencing datasets were analyzed to identify *PBK*-expressing cell populations. **Results**: Among 246 cases, 75 (30.5%) showed high *PBK* expression. High *PBK* expression was associated with lower TNM stage, absence of vascular invasion, and dMMR status. High-*PBK* tumors showed an immune-activated microenvironment, including increased CD4^+^, CD8^+^, and FOXP3^+^ T-cell infiltration, higher stromal PD-L1 expression, and higher tumor-infiltrating lymphocyte scores. Single-cell analysis indicated that *PBK* expression was enriched mainly in proliferative tumor epithelial cell populations. High *PBK* expression was associated with longer OS and RFS and remained an independent favorable prognostic factor in multivariate analysis. **Conclusions**: *PBK* expression in CRC is linked to proliferative tumor epithelial states, an immune-activated microenvironment, and favorable outcomes, supporting its utility as a prognostic biomarker.

## Simple Summary

Colorectal cancer shows large differences in patient outcomes, partly because tumors vary in their biological and immune characteristics. Identifying markers that reflect these differences is important for improving prognosis and treatment strategies. PDZ-binding kinase (PBK) is a protein involved in cell division and has been linked to cancer progression, but its clinical significance in colorectal cancer remains unclear. In this study, we examined *PBK* expression in tumor tissues from patients with colorectal cancer and analyzed its relationship with tumor features, immune cell infiltration, and patient survival. We found that tumors with high *PBK* expression were associated with a more active immune environment and better clinical outcomes. These findings suggest that *PBK* expression may help identify colorectal cancer patients with a favorable immune response and prognosis, providing useful information for future research and potential treatment stratification.

## 1. Introduction

Colorectal cancer (CRC) is one of the most common and lethal malignancies worldwide, with approximately 1.9 million new cases and 0.9 million deaths estimated in 2022 [1]. As population aging and lifestyle changes are expected to further increase the incidence of CRC, the identification of biologically based stratification and novel biomarkers remains an urgent clinical need.

In clinical practice, molecular features such as mismatch repair (MMR) deficiency and microsatellite instability (MSI), together with the TNM classification, are routinely used for the management of patients with cancer. Increasing evidence has also established the prognostic value of quantitative assessment of the tumor microenvironment (TME). Among tumor-associated immune cells, CD8^+^ T cells reflect cytotoxic antitumor activity, CD4^+^ T cells support adaptive immune responses, FOXP3^+^ regulatory T cells contribute to immunosuppression, and CD163^+^ macrophages represent an immunosuppressive myeloid compartment in the TME. In CRC, infiltration of CD4^+^ and CD8^+^ T cells, regulatory T cells (FOXP3^+^), and M2-type macrophages expressing CD163 has been reported to influence antitumor immune responses and clinical outcomes [2,3,4,5,6].

The immune checkpoint inhibitor pembrolizumab significantly prolonged progression-free survival compared with chemotherapy as a first-line treatment for dMMR (MSI-H) metastatic CRC, establishing a new standard of care [7]. In contrast, most pMMR tumors remain unresponsive, underscoring the need for novel predictive biomarkers from both tumor-intrinsic and immune perspectives. PD-L1 contributes to immune suppression within the TME and shows distinct expression patterns in tumor cells and tumor-infiltrating immune cells in CRC; however, its prognostic impact remains inconsistent across studies, likely reflecting differences in scoring approaches, cutoff values, and spatial heterogeneity [8,9,10,11,12].

PDZ-binding kinase (PBK), also known as T-LAK cell-originated protein kinase (TOPK), is a serine/threonine kinase involved in cell cycle regulation, DNA damage response, and inflammatory signaling [13]. *PBK/TOPK* is overexpressed in many malignancies and contributes to tumor proliferation, invasion, and metastasis through signaling pathways such as MAPK and FAK/Src. TOPK inhibitors, including HI-TOPK-032, OTS964/514, and SKLB-C05, have been shown to suppress tumor growth and metastasis and enhance radiosensitivity in preclinical models [14,15,16,17]. However, the clinical significance of *PBK* expression in CRC remains controversial. A large tissue microarray (TMA) study reported that diffuse TOPK overexpression was associated with poor prognosis in *KRAS*- or *BRAF*-mutated CRC [18], whereas several other studies demonstrated an inverse correlation between *PBK* expression and tumor stage, identifying *PBK* as an independent favorable prognostic factor [19,20,21]. These discrepancies may reflect differences in (i) detection methods (immunohistochemistry vs. mRNA-based assays), (ii) subcellular localization (nuclear, cytoplasmic, or total expression) and scoring criteria, (iii) molecular background (*KRAS*/*BRAF* mutations, MMR status (dMMR/pMMR)), and (iv) the evaluated components (tumor cells vs. immune cells).

To address these issues, a spatially resolved approach for detecting *PBK* transcripts in tumor cells and integrating them with immune context is required. In this study, *PBK* mRNA was assessed in formalin-fixed, paraffin-embedded sections using RNAscope-based RNA in situ hybridization (RNA-ISH), which provides in situ transcript detection at single-cell resolution [22]. Using serial TMA sections, we evaluated PBK expression together with immune markers and stromal PD-L1 to characterize the local immune context at the invasive front. Publicly available single-cell RNA sequencing (scRNA-seq) datasets can help validate *PBK* expression and localization in specific cell types.

We hypothesized that *PBK* expression in tumor epithelial cells is associated with an immune-activated tumor microenvironment and favorable clinical outcomes in colorectal cancer, and that *PBK* is primarily localized to a proliferative epithelial compartment. To test this hypothesis, we quantitatively assessed *PBK* expression in CRC tumor cells using spatially resolved RNA-ISH and analyzed its association with clinicopathological characteristics and patient prognosis. In the same cases, we evaluated CD4, CD8, FOXP3, CD163, and stromal PD-L1 by immunohistochemistry to characterize the immune profile of the tumor microenvironment. Finally, we analyzed publicly available single-cell RNA-seq data to confirm cell type-specific *PBK* expression and its predominant localization in proliferative epithelial cells.

## 2. Materials and Methods

### 2.1. Patients

This study included 305 patients with CRC treated surgically at Shinshu University Hospital between 2014 and 2022. All patients were monitored for a minimum follow-up period of 2 years. Tumor differentiation was assessed, and well-differentiated, moderately-differentiated, and poorly differentiated adenocarcinomas were included in the analysis. Following previously published criteria [23], well-differentiated and moderately-differentiated adenocarcinomas were classified as low-grade tumors, whereas poorly differentiated adenocarcinomas were categorized as high-grade tumors. Among the 305 patients, 59 were excluded for the following reasons: 42 were negative for the positive control (housekeeping gene) in the TMA and 17 cases had no tumor tissue at the primary site within a TMA. Ultimately, 246 patients with CRC were enrolled.

Clinical and pathological data, including patient age, sex, tumor differentiation, prognosis, lymph node involvement, vascular invasion, tumor-infiltrating lymphocytes (TILs), tumor location, and TNM classification, were extracted from medical records. Tumor staging and differentiation were defined following the eighth edition of the Union for International Cancer Control classification [24] and the fifth edition of the World Health Organization classification [25]. The levels of TILs in tumor regions were scored using a four-tier system: 0 (none), 1 (mild), 2 (moderate), and 3 (marked), as previously described [26]. TILs were assessed on hematoxylin and eosin–stained TMA sections in the tumor-associated stromal area at the invasive front. TIL scores were categorized as low (scores 0 and 1) or high (scores 2 and 3).

Overall survival (OS) was defined as the duration from the date of surgical resection to death or last follow-up. RFS was defined as the time from surgical resection to disease recurrence or the last follow-up without recurrence.

This study adhered to the ethical principles outlined in the Declaration of Helsinki and was approved by the Clinical Trial Review Committee of Shinshu University School of Medicine (approval number: 5836).

### 2.2. TMA Construction and Histopathology

Specimens were fixed in 10% or 20% neutral-buffered formalin and embedded in paraffin. For the construction of the TMA, blocks containing sufficient tumor tissue from the invasive front were selected from formalin-fixed paraffin-embedded tissue archives. Tissue cores (3 mm diameter) were punched out from each block using thin-walled stainless steel needles (Azumaya Medical Instruments Inc., Tokyo, Japan) and arrayed into a recipient paraffin block. Serial 4-µm-thick sections were cut from the TMA blocks, and one section was stained with hematoxylin and eosin for histological assessment.

### 2.3. IHC and Evaluation

IHC staining for CD4, CD8, FOXP3, and CD163 was performed on serial TMA sections. The staining was carried out using a fully automated staining system (BOND-III; Leica Biosystems, Newcastle, UK) with the following primary antibodies: CD4 (clone 4B12, ready-to-use; Leica Biosystems, Newcastle, UK), CD8 (clone C8/144B, ready-to-use; Leica Biosystems, Newcastle, UK), FOXP3 (clone 236A/E7, 1:100 dilution; Abcam, Cambridge, UK), and CD163 (clone 10D6, ready-to-use; Leica Biosystems, Newcastle, UK).

For the evaluation of CD4^+^, CD8^+^, and FOXP3^+^ T cells, three areas with the highest cell density were selected from each core, and cell counts per high-power field (10× ocular, 40× objective) were determined. The average of the three fields was calculated, and the median served as the cutoff to classify cases into low and high groups. For the evaluation of CD163, expression was evaluated semiquantitatively using the immunoreactivity score, which was calculated by multiplying the staining intensity score (0–3 scale) by the score for the percentage of positive cells (0–4 scale), as previously described [27]. Cases were classified into high and low CD163 expression groups using the median immunoreactivity score.

IHC staining of the TMA was also performed for mismatch repair proteins (MMR), including MLH1 (clone ES05, mouse monoclonal, 1:50), PMS2 (clone EP51, rabbit monoclonal, 1:40), MSH2 (clone FE11, mouse monoclonal, 1:50), and MSH6 (clone EP49, rabbit monoclonal, 1:50; Agilent Technologies, Santa Clara, CA, USA), using validated protocols [28]. A specimen was considered MMR-deficient if any of the four MMR proteins showed a complete lack of immunoreactivity.

PD-L1 expression was evaluated by IHC using a rabbit monoclonal anti-PD-L1 antibody (clone SP142, 1:100 dilution; Abcam, Cambridge, UK). PD-L1 evaluation was performed as previously described and was restricted to specimens containing more than 50 viable tumor cells [11]. PD-L1 staining of any intensity in tumor-associated stromal immune cells, including lymphocytes, macrophages, and dendritic cells, was considered positive. The percentage of PD-L1-positive immune cells within the tumor-associated stromal area was calculated irrespective of staining intensity and classified using a four-tier scoring system: 0 (<1%), 1 (≥1% to <5%), 2 (≥5% to <50%), and 3 (≥50%). Scores 0 and 1 were defined as low expression, and scores 2 and 3 were defined as high expression.

All histological features and staining results were independently evaluated by two experienced pathologists (T.U. and M.I.).

### 2.4. PBK RNA In Situ Hybridization

The detection of *PBK* mRNA was performed on unstained samples on tissue slides using the RNAscope™ 2.5 LS Probe–Hs-PBK (Cat. No. 551878; Advanced Cell Diagnostics, Newark, CA, USA) following the manufacturer’s instructions. Briefly, tissue sections were pretreated with heat and protease prior to hybridization as previously described [29]. Brown punctate dots observed in the nucleus or cytoplasm were considered positive signals. Standard Mm-PPIB (ACD-313902) was used as a positive control.

*PBK* expression was quantified under a 40× objective lens (BX53 microscope; Olympus Corporation, Tokyo, Japan) following the five-grade scoring system recommended by the manufacturer, as previously described for RNAscope scoring [30,31]: no staining (0), 1–3 dots/cell (1+), 4–9 dots/cell (2+), 10–15 dots/cell and/or <10% dots in clusters (3+), and >15 dots/cell and/or >10% dots in clusters (4+). Samples were classified into low *PBK* expression (grades 0, 1+, and 2+) and high *PBK* expression (grades 3+ and 4+).

### 2.5. Single-Cell RNA Sequencing Analysis of PBK Expression in CRC

Single-cell RNA sequencing analysis of *PBK* expression was conducted using a publicly available dataset (accession number: GSE132465) obtained from the NCBI Gene Expression Omnibus (GEO) database. This dataset comprised 23 tumor samples and 10 normal samples derived from CRC tissues [32]. Data processing and analysis were conducted using Seurat (v5.3.1; R Foundation for Statistical Computing, Vienna, Austria) in R (v4.5.0; R Foundation for Statistical Computing, Vienna, Austria). Raw count matrices were normalized to a total expression of 10,000 molecules per cell and subsequently scaled. Highly variable genes across cells were identified and utilized for principal component analysis to reduce dimensionality. To assess cellular similarities and perform clustering, the FindNeighbors and FindClusters functions were used. The visualization of cellular heterogeneity was achieved by applying uniform manifold approximation and projection using the RunUMAP function. Cell type annotation was performed based on canonical marker genes, and marker expression across annotated cell types was visualized as a heatmap (Figure 4e). The annotated cell types included epithelial cells (EPCAM, KRT8/18/19), T cells (CD3D, CD3E, TRAC), CD8 T cells (CD8A, CD8B), B cells (MS4A1, CD79A), plasma cells (MZB1, XBP1, JCHAIN), myeloid/monocytes (LYZ, LST1, S100A8/A9), endothelial cells (PECAM1, VWF, KDR), fibroblasts (COL1A1, COL1A2, DCN), and pericytes (RGS5, CSPG4, PDGFRB, MCAM). *PBK* expression was then assessed across annotated clusters.

### 2.6. Cell–Cell Communication Analysis

To investigate intercellular communication between *PBK*-high epithelial cells and other tumor microenvironmental cell types, CellChat analysis was performed using CellChat (version 1.6.1; R Foundation for Statistical Computing, Vienna, Austria) with the Seurat object described above [33]. The Epithelial_*PBK*hi cluster was defined as epithelial cells with high *PBK* expression in the scRNA-seq dataset, and these cells were used as the source population for CellChat analysis. The Epithelial_*PBK*hi cluster was designated as the ligand-expressing source. All other clusters were treated as potential receptor-expressing targets. Communication probabilities were inferred using the computeCommunProb function, and statistically significant ligand–receptor interactions were identified by permutation testing (*p* < 0.05). Aggregated pathway networks were visualized using the netVisual_aggregate function (circle layout), and pathway-level communication strength was summarized as the total communication probability across all target cell types. The six pathways showing the highest total communication probabilities were extracted for detailed visualization. Bootstrap resampling (*n* = 100) was performed to estimate the 95% confidence interval (CI) for each pathway’s total communication probability. Bubble plots were generated using netVisual_bubble to depict significant ligand–receptor pairs, with circle color indicating communication strength and size indicating *p*-value significance. *PBK*-high and *PBK*-low groups and differences (Δ*PBK* = *PBK*-high − *PBK*-low) were summarized as a heatmap to highlight pathway-level trends.

### 2.7. Statistical Analysis

Categorical variables were expressed as frequencies, and differences between subgroups were assessed using Fisher’s exact test. Cases with missing or unevaluable data were excluded on a per-variable basis. The Mann–Whitney *U*-test was used to compare immune cell infiltration levels (CD4^+^, CD8^+^, and FOXP3^+^ cells) between the *PBK* high-expression and low-expression groups. To visualize the distribution of immune cell counts, violin plots were generated using the ggplot2 package (version 4.0.1; R Foundation for Statistical Computing, Vienna, Austria). OS was analyzed in the entire cohort of 246 patients (stage 0–IV) and RFS analysis was limited to the 201 patients with non-metastatic (stage 0–III) disease. OS and RFS were estimated by the Kaplan–Meier method, and between-group comparisons were performed using the log-rank test. Prognostic factors were analyzed through univariate and multivariate Cox proportional hazards regression models. Statistical significance was defined as *p* < 0.05. All statistical analyses were performed using RStudio (version 2025.09.0+387; Posit Software, PBC, Boston, MA, USA) and EZR (Easy R, version 1.66; Jichi Medical University Saitama Medical Center, Saitama, Japan), a graphical user interface for R (The R Foundation for Statistical Computing, Vienna, Austria).

## 3. Results

### 3.1. Association Between PBK Expression and Clinicopathological Characteristics of Patients with CRC

**Figure 1:**
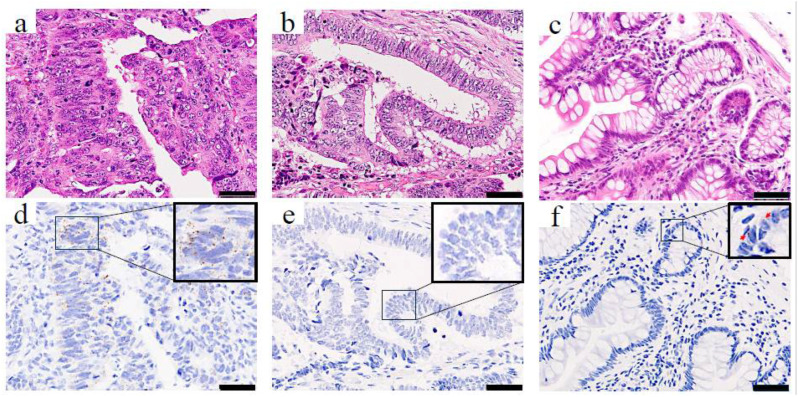
*PBK* expression in colorectal cancer and normal colonic epithelium visualized by RNA-ISH. H&E staining (top row) and RNA-ISH staining (bottom row) of (**a**,**d**) a *PBK*-high case, (**b**,**e**) a *PBK*-low case, and (**c**,**f**) normal colonic mucosa. In the *PBK*-high tumor (**d**), brown punctate RNA-ISH signals are clearly visible in the cytoplasm, whereas the *PBK*-low tumor (**e**) shows minimal staining. Weak *PBK* expression is observed in epithelial cells, and only faint *PBK* expression is detected in normal colonic epithelium (**f**). Red arrows indicate *PBK* signals. Scale bar: 50 µm.

To determine the clinical significance of *PBK* in CRC, we first assessed *PBK* expression in tumor samples from patients with CRC using RNA-ISH. *PBK* expression was evaluable in all 246 CRC tumor samples. *PBK* signals were detectable in 215 cases, whereas 31 cases showed no detectable *PBK* signal (score 0). 75 cases were classified as having high *PBK* expression (grade ≥ 3+). In *PBK*-high cases, *PBK* mRNA signals appeared as brown punctate dots predominantly in the cytoplasm of cancer cells (Figure 1a,d). PBK-low cases showed absent-to-low *PBK* signals (Figure 1b,e). Weak *PBK* signals were occasionally observed in epithelial cells of normal colonic mucosa (Figure 1c,f).

The associations between *PBK* expression and clinicopathological factors of patients with CRC are summarized in Table 1. High *PBK* expression was significantly more frequent in tumors located in the proximal colon and less frequent in those in the rectum (*p* = 0.003). High *PBK* expression was significantly associated with the absence of venous invasion (*p* = 0.002), absence of lymph node metastasis (*p* = 0.001), and early pathological stage (stage 0–II, *p* < 0.001). These findings indicate that *PBK*-high tumors exhibit less advanced pathological features. Lymphatic invasion tended to be less frequent in the *PBK*-high group, but the difference was of borderline significance (*p* = 0.052). No significant correlations were found between *PBK* expression and patient age (*p* = 0.268), sex (*p* = 0.329), or histological grade (*p* = 0.823).

### 3.2. Association Between PBK Expression and Immune-Related Parameters

**Figure 2:**
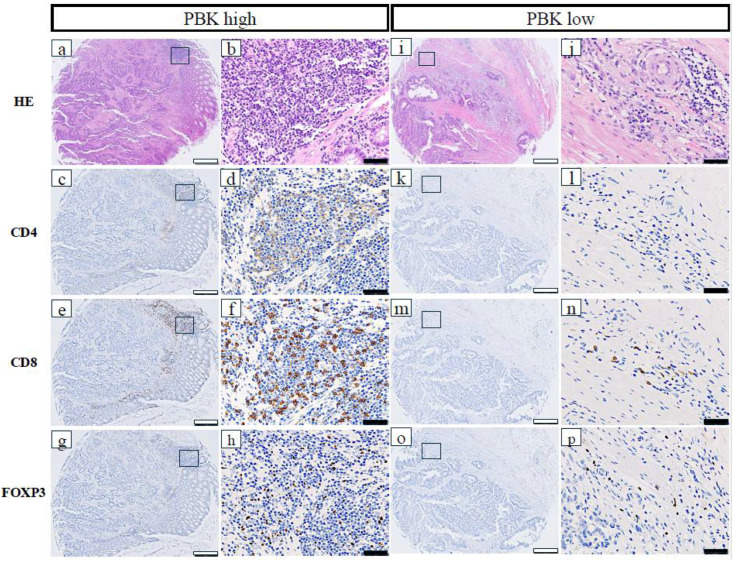
Representative immunohistochemical staining of CD4, CD8, and FOXP3 in *PBK*-high and *PBK*-low tumors. (**a**–**h**) *PBK*-high case; (**i**–**p**) *PBK*-low case. H&E, CD4, CD8, and FOXP3 staining was performed on serial tissue microarray sections. White scale bar: 500 µm; black scale bar: 50 µm.

The relationships between *PBK* expression and immune-related markers in patients with CRC are shown in Table 1. High *PBK* expression was significantly correlated with high CD4^+^ T-cell infiltration (*p* = 0.021), high CD8^+^ T-cell infiltration (*p* < 0.001), high FOXP3^+^ regulatory T-cell infiltration (*p* = 0.002), high PD-L1 expression in stromal immune cells (*p* < 0.001), and a high overall TIL score (*p* < 0.001). Additionally, dMMR status was significantly more frequent in *PBK*-high cases (*p* = 0.018). No significant association was observed between *PBK* and CD163 expression (*p* = 0.289). Representative images of CD4^+^, CD8^+^, and FOXP3^+^ cells in *PBK*-high and *PBK*-low tumors are shown in Figure 2.

**Figure 3:**
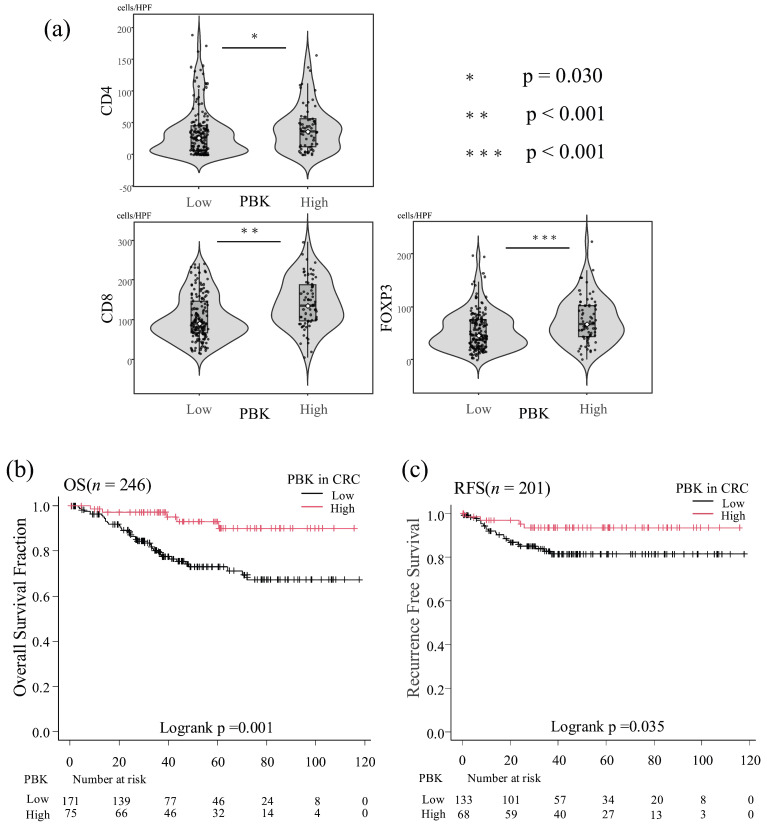
Association between *PBK* expression, tumor-infiltrating T cells, and patient prognosis. (**a**) Violin plots showing the distribution of CD4^+^, CD8^+^, and FOXP3^+^ T-cell counts in patients with colorectal cancer stratified by *PBK* expression status. Boxes represent interquartile ranges with medians; whiskers indicate 1.5× interquartile range. Mann–Whitney U tests demonstrated significantly higher infiltration of CD4^+^ (*p* = 0.030), CD8^+^ (*p* < 0.001), and FOXP3^+^ (*p* < 0.001) T cells in *PBK*-high tumors. (**b**) Kaplan–Meier curves of overall survival (OS) of patients with colorectal cancer stratified by *PBK* expression (*n* = 246). Patients with high *PBK* expression showed significantly better OS than those with low *PBK* expression (log-rank *p* = 0.001). (**c**) Kaplan–Meier curves of recurrence-free survival (RFS) in patients with non-metastatic colorectal cancer (*n* = 201). High *PBK* expression was associated with longer RFS compared with low *PBK* expression (log-rank *p* = 0.035).

Immune cell counts were analyzed as continuous variables using the Mann–Whitney U test. *PBK*-high tumors showed significantly higher infiltration of CD8^+^ T cells (*p* < 0.001), CD4^+^ T cells (*p* = 0.030), and FOXP3^+^ T cells (*p* < 0.001) compared with PBK-low tumors (Figure 3a).

### 3.3. Association Between PBK Expression and OS

In the Kaplan–Meier analysis of the overall group of 246 patients, high *PBK* expression was significantly associated with longer OS compared with low *PBK* expression (log-rank *p* = 0.001; Figure 3b).

In univariate Cox regression, high *PBK* expression was a significant protective factor against mortality (HR = 0.25, 95% CI 0.10–0.62, *p* = 0.003). Multivariate analysis adjusting for age, sex, histological type, vascular invasion, lymph node metastasis, and TNM stage confirmed *PBK* as an independent favorable prognostic factor for OS (HR = 0.28, 95% CI 0.11–0.72, *p* = 0.008; Table 2).

### 3.4. Association Between PBK Expression and RFS

In the 201 patients with non-metastatic CRC (stage 0–III), Kaplan–Meier curves demonstrated that *PBK*-high tumors were associated with significantly longer RFS than *PBK*-low tumors (log-rank *p* = 0.035; Figure 3c).

In univariate Cox analysis, high *PBK* expression correlated with reduced recurrence risk (HR = 0.34, 95% CI 0.12–0.98, *p* = 0.045). However, this association was not statistically significant after multivariate adjustment for age, sex, lymphatic invasion, and TNM stage (*p* = 0.146; Table 3).

### 3.5. scRNA-Seq Analysis of PBK Expression in CRC

**Figure 4:**
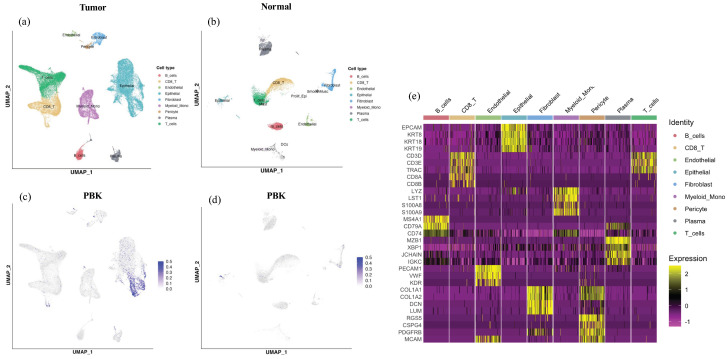
scRNA-seq analysis of *PBK* expression in colorectal cancer. UMAP plots showing cell clusters in tumor (**a**) and normal (**b**) tissues, including epithelial, T, B, mast, myeloid, and stromal cells. *PBK* expression was visualized in tumor (**c**) and normal (**d**) tissues, showing predominant localization in epithelial clusters in tumors and minimal expression in normal tissues. (**e**) Heatmap showing expression of canonical marker genes used for cell type annotation in the colorectal cancer scRNA-seq dataset. Cells are grouped by the annotated cell types shown above the heatmap. Color indicates scaled expression.

Analysis of a publicly available scRNA-seq dataset (GSE132465) revealed that *PBK* expression was predominantly localized in the epithelial cell clusters within tumor tissues. In contrast, immune and stromal populations such as T cells, B cells, mast cells, myeloid cells, and fibroblasts exhibited minimal or no *PBK* expression (Figure 4a–d). In normal colonic samples, *PBK* expression was generally low, with only scattered weak signals detected in epithelial clusters. Cell type annotations were supported by canonical marker gene expression patterns summarized in a heatmap (Figure 4e)

### 3.6. Cell–Cell Communication Analysis by CellChat

**Figure 5:**
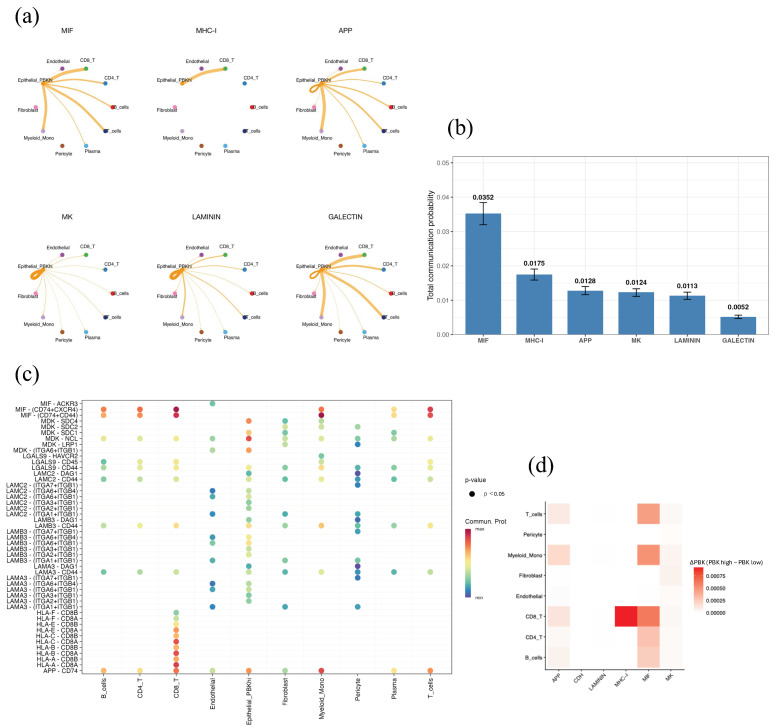
Cell–cell communication analysis of *PBK*-high epithelial cells by CellChat. (**a**) Circle plot of major signaling networks originating from *PBK*-high epithelial cells (Epithelial_*PBK*_hi). Epithelial_*PBK*_hi was defined as the epithelial subset showing high *PBK* expression in the scRNA-seq dataset. The top six enriched pathways are MIF, MHC-I, APP, MK, LAMININ, and GALECTIN. (**b**) Bar plot showing the total communication probability (mean with 95% CI) across pathways, highlighting MIF and MHC-I as dominant. “Total communication probability” represents the overall inferred strength of pathway-level communication from Epithelial_*PBK*_hi cells to all target cell types. (**c**) Bubble plot of significant ligand–receptor pairs (*p* < 0.05), including MIF–(CD74 + CXCR4), MHC-I–(HLA-B–CD8A), and LAMININ–(LAMB3–ITGA6 + ITGB1). Bubble color indicates communication probability. (**d**) Heatmap showing differential communication (Δ*PBK* = *PBK*-high − *PBK*-low), with higher MIF and MHC-I activity in *PBK*-high tumors. Δ*PBK* was calculated as the difference between *PBK*-high and *PBK*-low epithelial subsets.

CellChat analysis was performed to explore ligand–receptor interactions between *PBK*-high epithelial cells (Epithelial_*PBK*_hi), defined as the epithelial subset showing high *PBK* expression in the scRNA-seq dataset, and other cell populations in the tumor microenvironment. Signaling pathways were ranked according to the total communication probability, which represents the overall inferred strength of pathway-level communication from Epithelial_*PBK*_hi cells to all target cell types. Among signaling pathways ranked by total communication probability, the MIF and MHC-I pathways showed the highest levels of intercellular activity, followed by APP, MK, LAMININ, and GALECTIN (Figure 5a,b).

*PBK*-high epithelial cells interacted extensively with CD8^+^ and CD4^+^ T cells, B cells, monocytes, and fibroblasts through specific ligand–receptor pairs, such as MIF–(CD74 + CXCR4) and MHC-I–(HLA-B–CD8A) (Figure 5c).

We compared *PBK*-high and *PBK*-low epithelial subsets, and difference analysis (Δ*PBK* = *PBK*-high − *PBK*-low) revealed relatively stronger communication through the MIF and MHC-I pathways, particularly highlighting enhanced MHC-I–CD8^+^ T-cell interactions in *PBK*-high tumors (Figure 5d).

## 4. Discussion

In this study, we examined the clinical significance of *PBK* expression and its association with the tumor immune microenvironment in CRC using RNA-ISH. By combining spatially resolved *PBK* mRNA detection with immune profiling and public scRNA-seq analyses, we interpret *PBK*-high CRC as a proliferative epithelial state embedded within an immune-activated microenvironment, which may help explain inconsistent prognostic reports in prior *PBK/TOPK* studies by emphasizing assay- and context-dependent interpretation.

PBK, also known as TOPK, is a MAPKK-like serine/threonine kinase whose expression peaks during the G2/M phase of the cell cycle and that contributes to histone H3 phosphorylation and the DNA damage response [13,15,34]. In CRC, higher *PBK/TOPK* expression has been reported to correlate with favorable outcomes in some cohorts, consistent with our findings [19,20]. In our cohort, *PBK*-high status remained independently associated with overall survival, whereas the association with recurrence-free survival was attenuated after adjustment for established clinicopathological factors. This attenuation may reflect confounding by TNM stage and the limited number of recurrence events, which may reduce power to detect independent effects on RFS. *PBK/TOPK* shows variable prognostic associations in CRC, and these differences likely reflect heterogeneity in assay modality (protein- vs. transcript-based), scoring and cutoff definitions, and consideration of localization, as well as tumor background and immune context (e.g., dMMR/pMMR status and immune infiltration).

The strong parallel between high *PBK* expression and infiltration of CD4^+^ and CD8^+^ T cells in CRC observed in our study suggests that *PBK* expression may reflect an immune-activated TME. In CRC, tumor-infiltrating T-cell density is a robust prognostic factor and forms the basis of the clinically implemented “Immunoscore” [35]. Previous analyses of public datasets have also demonstrated that high *PBK* expression correlates positively with CD8^+^ T-cell and natural killer cell infiltration, as well as high tumor mutational burden (TMB) and dMMR status, indicating enhanced cytotoxic immune activity [19]. Moreover, comprehensive transcriptomic analyses have shown that *PBK* expression is tightly linked to cell cycle and mitotic pathways and is positively associated with immune checkpoint-related genes [14]. These findings support an interpretation that *PBK*-high tumors align with an IFN-γ-associated, immune-active state potentially linked to tumor-intrinsic proliferative and stress programs, although causal mechanisms require functional validation.

The association between *PBK* and FOXP3^+^ regulatory T cells also deserves consideration. While FOXP3^+^ regulatory T cells generally exert immunosuppressive functions, several studies have suggested that FOXP3^+^ T-cell infiltration may contribute to favorable prognosis in CRC by maintaining mucosal immune homeostasis and limiting excessive inflammation [36,37]. In our cohort, *PBK* expression was not correlated with CD163, a marker of M2-like macrophages, indicating that *PBK* expression reflects a T-cell-dominant, rather than macrophage-dominant, inflammatory TME.

The positive correlation between *PBK* and PD-L1 expression in stromal immune cells is also noteworthy. In CRC, stromal (immune cell–associated) PD-L1 is often interpreted as an IFN-γ–induced readout of ongoing antitumor immune activity, whereas tumor-cell PD-L1 is more heterogeneous and less consistently linked to clinical outcomes; therefore, we focused our evaluation on stromal PD-L1. While PD-L1 expression on tumor cells is often associated with poor prognosis, PD-L1 expression in stromal immune cells, which is typically IFN-γ-induced, has been linked to improved clinical outcomes [38]. In our cohort, PD-L1 overexpression was a favorable prognostic factor, and its elevation in *PBK*-high tumors is consistent with an IFN-γ-dependent immune response. Clinically, stromal PD-L1 in *PBK*-high tumors may therefore act as a readout of pre-existing immune engagement rather than tumor-intrinsic immune escape.

High *PBK* expression was frequently observed in dMMR (MSI-H) tumors. dMMR CRCs are characterized by a high TMB and abundant neoantigen production, leading to marked infiltration of CD8^+^ T cells and IFN-γ-induced PD-L1 expression [39,40]. Recent studies have reported that *PBK* expression positively correlates with MSI and the expression of immune checkpoint-related genes [19]. Our results are consistent with these observations, suggesting that *PBK*-high tumors reflect a highly immunogenic phenotype typical of dMMR CRC. The predominance of *PBK* expression in right-sided tumors also aligns with previous findings that dMMR and immune-rich transcriptional signatures are more frequent in the right colon [41,42].

Our scRNA-seq analysis revealed that *PBK* expression was mainly restricted to epithelial tumor cell clusters, with minimal expression in immune or stromal cells. Furthermore, CellChat analysis identified the MIF (macrophage migration inhibitory factor) and MHC-I signaling pathways as the major ligand–receptor routes associated with *PBK*-high epithelial cells. MIF participates in tumor inflammation and immune activation by engaging CD74 and CXCR4 to promote interactions with T cells and macrophages [43]. The MHC-I pathway, in contrast, reflects antigen-presenting capability and cytotoxic T-cell activation. Thus, *PBK*-high CRCs may exhibit enhanced antigen presentation and cytotoxic T-cell engagement through MHC-I–related programs, consistent with prior hypotheses [19]. Additionally, activation of the LAMININ pathway suggests potential remodeling of the extracellular matrix and epithelial–stromal crosstalk within the TME [44]. Together, these findings indicate that *PBK* expression in tumor cells may amplify immune activation through intercellular communication networks, consistent with our pathological and clinical observations. Nonetheless, CellChat analysis is based on transcriptional inference, and experimental validation using coculture systems or spatial transcriptomics will be required to confirm these signaling interactions.

The biological role of *PBK* may extend beyond tumor cell proliferation, influencing the immune milieu and therapeutic response. Recent CRC studies suggest that tumor-intrinsic proliferative programs can be prognostically and therapeutically relevant; this supports a context-dependent interpretation of growth-associated markers [45]. The *PBK* inhibitor SKLB-C05 has been shown to suppress tumor growth and metastasis in CRC models [17], and another selective inhibitor, HI-TOPK-032, enhanced natural killer cell cytotoxicity and antitumor immunity in other cancer models [46]. These observations support *PBK/TOPK* as a biologically relevant node linking proliferative programs to therapeutic vulnerability, while our clinical data primarily position *PBK* expression as a marker of an immune-active tumor state rather than evidence of direct immunotherapy modulation.

This study has several limitations. This was a retrospective, single-center analysis, and functional characterization of immune cell states (e.g., activation or exhaustion markers) and *PBK* phosphorylation or subcellular localization was not performed. Because *PBK* was quantified at the mRNA level by RNA-ISH, transcript abundance may not directly reflect *PBK* protein expression or kinase activity. Our immune profiling relied on immunohistochemistry; therefore, associations between *PBK* mRNA and immune protein markers should be interpreted as correlative rather than mechanistic. This also applies to PD-L1 staining, which is influenced by antibody clone selection; the SP142 clone preferentially highlights immune-cell PD-L1 and may underestimate tumor-cell PD-L1, which may limit comparability with studies using other clones or tumor-cell-focused scoring. Furthermore, the scRNA-seq data were derived from a public dataset and analyzed in a descriptive manner. Pathological evaluation was confined to the tumor invasive front and did not encompass whole-tumor sections. Future studies incorporating in-house scRNA-seq or spatial transcriptomic analyses will be necessary to elucidate the functional diversity of immune cell subsets and the spatial dynamics of *PBK*-high CRCs.

## 5. Conclusions

*PBK* is an independent prognostic factor for overall survival in colorectal cancer and is associated with an immune-activated tumor microenvironment. Our findings indicate that *PBK* expression reflects a proliferative epithelial state within an immune-active context, while its biological role and potential relevance to treatment sensitivity require prospective and functional validation.

## Figures and Tables

**Table 1:** Association between *PBK* expression and clinicopathological factors in patients with colorectal cancer (*n* = 246).

Factors		*PBK* Expression		*p*-Value
	Total	Low (*n* = 171)	High (*n* = 75)	
**Age**				**0.268**
<70 years	112	82	30	
≥70 years	134	89	45	
**Sex**				**0.329**
Male	143	103	40	
Female	103	68	35	
**Histological grade**				**0.823**
Low	221	154	67	
High	25	17	8	
**Lymphatic invasion**				**0.052**
Present	119	90	29	
Absent	127	81	46	
**Venous invasion**				**0.002**
Present	171	130	41	
Absent	75	41	34	
**Location**				**0.003**
Proximal	63	33	30	
Distal	75	55	20	
Rectal	108	83	25	
**LN metastasis**				**0.001**
Present	117	93	24	
Absent	129	78	51	
**TNM stage**				***p* < 0.001**
0–II	124	74	50	
III–IV	122	97	25	
**CD4**				**0.021**
High	111	70	41	
Low	120	93	27	
**CD8**				***p* < 0.001**
High	121	70	51	
Low	116	96	20	
**FOXP3**				**0.002**
High	123	75	48	
Low	114	91	23	
**CD163**				**0.289**
High	176	121	55	
Low	48	37	11	
**TIL**				***p* < 0.001**
High	124	70	54	
Low	122	101	21	
**MMR status**				**0.018**
dMMR	24	11	13	
pMMR	220	158	62	
**PD-L1**				***p* < 0.001**
Low	163	133	30	
High	78	36	42	

LN, lymph node; TNM, tumor–node–metastasis; TIL, tumor-infiltrating lymphocyte; dMMR, deficient mismatch repair; pMMR, proficient mismatch repair.

**Table 2:** Univariate and multivariate analyses of overall survival factors in patients with CRC.

Factors	Univariate Analysis					Multivariate Analysis				
	HR	95 %CI			*p*-Value	HR	95 %CI			*p*-Value
Age ≥ 70 years vs. <70 years	1.12	0.62	-	2.02	0.719	1.29	0.69	-	2.39	0.423
Sex: male vs. female	1.18	0.65	-	2.18	0.580	1.45	0.80	-	2.63	0.226
Histological grade: low vs. high	2.12	0.89	-	5.06	0.0918					
Lymphatic invasion: absent vs. present	3.96	1.96	-	8.02	***p* < 0.001**	3.56	1.76	-	7.23	***p* < 0.001**
Venous invasion: absent vs. present	1.67	0.80	-	3.47	0.172					
TIL low vs. high	0.26	0.12	-	0.53	***p* < 0.001**					
CD4 low vs. high	0.79	0.43	-	1.48	0.476					
CD8 low vs. high	0.56	0.30	-	1.06	**0.073**					
FOXP3 low vs. high	0.31	0.16	-	0.61	***p* < 0.001**					
CD163 low vs. high	1.08	0.50	-	2.35	0.843					
MMR status (dMMR vs. pMMR)	0.45	0.11		1.85	0.266					
PD-L1 low vs. high	0.26	0.10		0.65	**0.004**					
LN metastasis: absent vs. present	3.09	1.59	-	6.00	***p* < 0.001**					
TNM stage: 0–II vs. III–IV	3.60	1.78	-	7.28	***p* < 0.001**	1.96	0.90	-	4.28	0.091
*PBK* expression: low vs. high	0.25	0.10	-	0.62	**0.003**	0.28	0.11	-	0.72	**0.008**

HR, hazard ratio; TIL, tumor-infiltrating lymphocyte; CD4, cluster of differentiation 4; CD8, cluster of differentiation 8; FOXP3, forkhead box P3; CD163, cluster of differentiation 163; PD-L1, programmed death-ligand 1; dMMR, deficient mismatch repair; pMMR, proficient mismatch repair; LN, lymph node; TNM, tumor–node–metastasis.

**Table 3:** Univariate and multivariate analyses of recurrence-free survival factors in patients with CRC.

Factors	Univariate Analysis					Multivariate Analysis				
	HR	95 %CI			*p*-Value	HR	95 %CI			*p*-Value
Age ≥ 70 years vs. <70 years	1.15	0.52	-	2.55	0.738	1.40	0.62	-	3.12	0.415
Sex: male vs. female	1.03	0.47	-	2.28	0.935	1.13	0.51	-	2.49	0.766
Histological grade: low vs. high	0.84	0.20	-	3.57	0.814					
Lymphatic invasion: absent vs. present	2.62	1.16	-	5.93	**0.021**	1.26	0.50	-	3.19	0.629
Venous invasion: absent vs. present	1.74	0.69	-	4.35	0.239					
TIL low vs. high	0.83	0.38	-	1.82	0.645					
CD4 low vs. high	1.12	0.49	-	2.53	0.792					
CD8 low vs. high	0.88	0.40	-	1.96	0.759					
FOXP3 low vs. high	0.98	0.44	-	2.21	0.962					
PD-L1 low vs. high	0.30	0.10	-	0.86	0.025					
CD163 low vs. high	1.08	0.40	-	2.90	0.887					
TNM stage: 0–II vs. III	5.41	2.16	-	13.55	***p* < 0.001**	5.41	2.16	-	13.55	***p* < 0.001**
*PBK* expression: low vs. high	0.34	0.12	-	0.98	**0.045**	0.45	0.15	-	1.32	0.146

HR, hazard ratio; TIL, tumor-infiltrating lymphocyte; CD4, cluster of differentiation 4; CD8, cluster of differentiation 8; FOXP3, forkhead box P3; CD163, cluster of differentiation 163; PD-L1, programmed death-ligand 1; TNM, tumor–node–metastasis.

## Data Availability

The data presented in this study are available on request from the corresponding author. The data are not publicly available due to ethical restrictions.
